# The Future of St. Katharine's Hospital

**Published:** 1914-03-07

**Authors:** 


### THE FUTURE OF ST. KATHARINE'S HOSPITAL.

On Tuesday last, the Local Government Committee presented to the London County Council a report on the scheme for the regulation of St. Katharine's Hospital, an institution whose removal, both practical and spiritual, from the East End, has provoked considerable controversy. At the Council's meeting early in February it was stated that a new scheme would shortly be presented, by which the large finances of the institution would be made available for a college in East London, where women students could be trained in health work, and qualified women could reside. The report was adopted.

This scheme may be summarised as follows: Queen Alexandra, the patron, has determined that "the benefit of the foundation of St. Katharine's shall be restored to the poor of the East of London; and, secondly, that neither shall any person now on the foundation suffer change, nor shall its character or buildings be secularised." So near the old site of "St. Katharine-by-the-Tower" a college to be known as "The College of St. Katharine-by-the Tower" will be established (1) to provide duly qualified resident health workers, defined as women possessing public health and nursing certificates and maternity experience, who will work under the control of the medical officers of health of the borough councils, to whom, through the principal, they will send reports; and (2) to train women students, similarly qualified, but without practical experience in such work.

The new rules further provide that the office of master (a post created in 1878 at a salary of £800 a year and now vacant) be not filled, one of the brothers, of whom there are three receiving £300 a year, will continue to perform the services in the chapel at Regent's Park as acting master, and receive £400 a year and a house. The three sisters, also receiving £300 a year, will continue, but vacancies will not be filled. Of the ten extern sisters, and ten bedesmen and bedeswomen, no bedesmen or bedeswomen now appear to survive; and there are only three extern sisters (ladies in straitened circumstances who receive £100 a year each). When a vacancy occurs, Queen Alexandra may appoint two qualified extern sisters with stipends of £50 a year each. The school in Regent's Park will be carried on as before till the end of the current school year, and the school staff will be pensioned or given gratuities. It is anticipated, in view of the recent and prospective reductions in the establishment, that a considerable sum will be available for the new college, but the other functions are to be a first charge on the funds.

To make the present position clear a brief *résumé* of the history of the "Royal Hospital and the Chapel of St. Katharine-near-the-Tower" may be given, of which a capital outline appeared in the *Daily Telegraph* of February 7. Queen Matilda, Stephen's Consort, founded it in 1148, in memory of her son and daughter. Eleanor, wife of Henry III., dissolved the original foundation, and ordained that it should comprise a master, three brothers, three sisters, and certain bedeswomen, and that six poor children should be fed, clothed, and receive certain perquisites, while other sums were to be distributed. This began its life as a charity in the modern guise of a philanthropic institution. Queen Eleanor confirmed the founder's enactment that the patronage of the hospital should remain in the hands of the Queens of England for ever. Philippa, wife of Edward III., added to its endowments. It escaped secularisation at the dissolution of the monasteries. Under Queen Anne the Lord Chancellor, as the result of a Charter of 1445 which placed the hospital entirely under the jurisdiction of this official, decreed that the brothers should be in Holy Orders, and that a school for thirty-five boys and fifteen girls be set up. In the eighteenth century its buildings were largely pulled down, and in 1825 the company formed to construct St. Katharine's Dock wanted the site of the old church which still lingered. This was conceded, and the Regent's Park buildings were raised. In 1871 a Royal Commission inquired into diverting some at least of its large revenues back again to the East End, but nothing happened till the County Council, after several efforts, succeeded in bringing the question forward during the past year.

